# Deductive Biocomputing

**DOI:** 10.1371/journal.pone.0000339

**Published:** 2007-04-04

**Authors:** Jeff Shrager, Richard Waldinger, Mark Stickel, J.P. Massar

**Affiliations:** 1 Department of Plant Biology, Carnegie Institution of Washington, Stanford, California, United States of America; 2 Artificial Intelligence Center, SRI International, Menlo Park, California, United States of America; 3 Berkeley, California, United States of America; Northeastern University, United States of America

## Abstract

**Background:**

As biologists increasingly rely upon computational tools, it is imperative that they be able to appropriately apply these tools and clearly understand the methods the tools employ. Such tools must have access to all the relevant data and knowledge and, in some sense, “understand” biology so that they can serve biologists' goals appropriately and “explain” in biological terms how results are computed.

**Methodology/Principal Findings:**

We describe a deduction-based approach to biocomputation that semiautomatically combines knowledge, software, and data to satisfy goals expressed in a high-level biological language. The approach is implemented in an open source web-based biocomputing platform called BioDeducta, which combines SRI's SNARK theorem prover with the BioBike interactive integrated knowledge base. The biologist/user expresses a high-level conjecture, representing a biocomputational goal query, without indicating how this goal is to be achieved. A subject domain theory, represented in SNARK's logical language, transforms the terms in the conjecture into capabilities of the available resources and the background knowledge necessary to link them together. If the subject domain theory enables SNARK to prove the conjecture—that is, to find paths between the goal and BioBike resources—then the resulting proofs represent solutions to the conjecture/query. Such proofs provide provenance for each result, indicating in detail how they were computed. We demonstrate BioDeducta by showing how it can approximately replicate a previously published analysis of genes involved in the adaptation of cyanobacteria to different light niches.

**Conclusions/Significance:**

Through the use of automated deduction guided by a biological subject domain theory, this work is a step towards enabling biologists to conveniently and efficiently marshal integrated knowledge, data, and computational tools toward resolving complex biological queries.

## Introduction

### Background

Biologists must increasingly conduct computational analyses across integrated biological knowledge and data [1], but biologists who cannot program are relegated to searching in knowledge bases and carrying out canned computations that have been programmed by others. To conduct novel computations—ones for which no canned solution exists—biologists often hire programmers, but this is either expensive or hit-and-miss (or both), and usually results in overly specific one-off solutions. Moreover, putting non-biologist programmers between biologists and biocomputation removes the biologist from the details of the computation [2], which makes biologists nervous about the correctness of the results—as well it should. Unlike physics, there is no clear categorization within biology of, for example, theoretical vs. experimental vs. computational biologists whose practitioners could naturally collaborate. As a result, it is often nearly impossible for the authors of papers (usually biologists) to accurately and completely describe the computational methods used to solve a given biological problem. Although this will certainly change as computational techniques become standardized and biologists learn to program—indeed a whole new species called “computational biologist” is rapidly evolving—it would be useful if biologists had “intelligent” tools that, in a sense, “understand” biology, could help the biologists conduct analyses, and could explain how the analyses were accomplished, all expressed in terms that are natural to the biologists.

The present paper explores just such an “intelligent” paradigm that we call *deductive biocomputing*. In our paradigm biologists/users express queries as goals in high-level near-natural terms. Others—usually biologists who are more computationally savvy working with programmers—provide instructions to the platform about how to satisfy those goals. Automatic methods then put these together to satisfy the goals and to explicitly record how the answers were discovered. This is analogous to the widely studied problem of automatic program generation [3], [4] in which a specification for a program is expressed in a high-level language and then translated into an actual program that implements the specification. (Although fully general automatic program synthesis is an unsolved problem, the present query resolution problem is more tractable because we are looking for an answer to a specific query, not a procedure that will answer an entire class of queries. In particular, because the inputs to the query are generally known in advance, we can evaluate tests and unwind loops at proof-time, this avoiding having to automatically generate conditional branches and iterative or recursive loops.)

### The deductive approach to biocomputation

Engineers approach complex knowledge-based goals in three general ways: (a) the “procedural” approach, (b) the “relational” approach, and (c) the “deductive” approach. In the procedural approach (a) one specifies each step required to reach the goal, down to the level at which the steps are primitives in the domain language. Such procedures often involve search in knowledge bases, generally traversing the structures that represent complex knowledge, transforming data, and conducting specific calculations along the way. Usually writing programs of this sort is beyond the skill of biologists.

In the relational approach (b) queries are written such that the complex inner loops (searching over records) are implicit; for example: “Select genes (g1) of organism1 with the word ‘photosynthesis’ in their annotation field, and where there is a gene (g2) in organism2 such that g1 and g2 are orthologous.” Such queries can be directly executed by the relational database engine, thus simplifying the programming process. Still, in order to make use of this approach in complex analyses the biologist must know a great deal about how the knowledge bases are structured, and what tools are available.

In the deductive approach (c), the biologist specifies a high-level goal, usually without knowing how it will be satisfied, and a runtime executor (called a *theorem prover* for reasons that will become clear below) does whatever work is required to satisfy the goal. One might, for example, ask: “Find photosynthetic orthologs between organism1 and organism2.” Whereas the relational approach goes beyond the procedural approach by leaving loop optimization to the computer, the deductive approach goes beyond even the relational approach by avoiding the necessity of describing an algorithm at all. No computation is explicitly described; instead, the query is expressed in declarative, problem-appropriate terms; it is the job of the theorem prover to bridge the gap between the query-level concepts and the computational ones.

Leaving method determination to the computer is a significant though difficult advance; whereas the implicit search carried out by the relational database engine requires knowledge of how to execute and optimize a certain class of algorithms (complex loops over relational databases), the deductive approach requires that the computer have additional understanding of how aspects of the knowledge base are connected to one another, and how to use these connections in service of high-level goals. The computer must, in a sense, “understand” how the knowledge is organized, and how it relates to high-level goals. Generally the knowledge-base designer provides such information to the theorem prover in what we shall call a *subject domain theory*. Rather than representing one-off methods, this knowledge can be expressed modularly so that it can be applied to a wide array of different goals. Through this specification of a subject domain theory and application of a general theorem prover, the end-user biologist—the person at the top who needs the results of the computation to begin with—is relying on the knowledge-base designers and the theorem prover to do the heavy lifting. At the end of this paper we will see that in addition to providing great power to the biologist-user, deductive biocomputing affords a number of significant advantages over either task-specific programs or relational queries, including a coherent way of recording where results come from (called *provenance*). Also, one can nearly (but not quite) get to true natural language programming, in which the biologist can ask a question in plain language and efficiently obtain both results and a detailed explanation of how they were calculated. But there are also efficiency limitations to this technique, and special requirements in terms of the consistency of the symbols (names) used in the subject domain theory. We will return to these points in the discussion.

The core of a deductive approach is the subject domain theory, an ontology comprising definitions of domain concepts, descriptions of the capabilities of available resources including data, knowledge, and tools, and the background knowledge necessary to relate these to high-level queries. While the word *ontology* is sometimes taken to mean a description of vocabulary and taxonomy of the domain, we mean here a formal axiomatic theory that defines domain concepts and relates them to one another. We use the word *axiom* to include what is often called a *rule* in the Semantic Web or Expert Systems literature. We employ full first-order logic as the representation language of the subject domain theory. (Readers familiar with theorem proving technologies will correctly recoil at the undecidability of full first-order logic. We address this issue in some detail in the Discussion section of the paper.)The query, or question to be answered, is also expressed in the language of logic; specifically it is phrased as a conjecture whose validity is to be proved by an automatic proof in the subject domain theory (thus the term *theorem prover*). The axioms of the subject domain theory allow the query conjecture to be transformed and decomposed into subgoals, leading eventually (one hopes) to a proof. If a proof is found, answer-extraction techniques are applied to the proof to yield an answer [3], [4]. If more than one proof is found, more than one answer may be extracted.

## Method

### BioDeducta = SNARK+BioBike

While the applicability of the deductive approach is independent of any particular implementation, our prototype implementation, called BioDeducta, is built largely from existing components. The theorem prover is SRI's reasoner SNARK [5]. The query and subject domain theory are formulated in SNARK's logical language. SNARK has a procedural-attachment mechanism that allows one to consult external resources while a proof is in progress, and a mechanism for extracting answers to queries from discovered proofs. SNARK uses a “sorted logic”, in which each entity bears a syntactic indication of what “sort”, or type, of object it is.

Biology-specific data, knowledge, and software resources are drawn from the BioBike environment [2], an integrated biological data and knowledge repository and biology-specific programming environment. BioBike provides integrated access to a number of knowledge sources, including the Gene Ontology (GO) [6], Kegg (www.genome.jp/kegg/), a BioCyc Database [7] for Cyanobacteria, and biological literature, as well as important biological software tools such as Meme, BLAST, R, and Clustal. BioBike is built on top of the KnowOS (Knowledge Operating System) [8], which embeds the biological knowledge and data in a frame-based programmable knowledge environment, and provides web-based access to it, as well as the ability to call out to remote resources over the web. The KnowOS includes SNARK as a built-in facility, and SNARK procedural attachments link symbols of the SNARK subject domain theory to BioBike functions. Both SNARK and BioBike are written entirely in Common Lisp, and are offered as open-source freeware. Moreover, there is a fully functional BioBike demo server, including SNARK, on which the examples in this paper can be tested, and wherein users can freely develop new BioDeducta applications of their own design (see www.biobike.org).

### The “hli” problem

We here illustrate the BioDeducta approach with an extended, realistic example. The cyanobacterium subspecies Procholorococcus is widely distributed in the world's oceans, and plays a critical role both in the marine ecosystem and in the global carbon cycle [9]. A problem of interest to biologists is how these organisms are adapted to their environmental niches. Bahya et al. [10] studied the genomic differences among many strains of the cyanobacteria with respect to their adaptation to niches of differing levels of light and nutrients. Among the Procholorococci, Procholorococcus sp. strain Med4 (aka. ProMed4 or Med4) is adapted to high light, living in the upper part of the ocean, whereas Procholorococcus sp. strain MIT9313 (aka. Pro9313) is adapted to lower light, living in somewhat deeper waters (although still in the “euphotic zone” where there is some available sunlight). Bhaya et al. were interested in which proteins (and their genes) are involved in this adaptation—that is those one might call the *high light adaptive genes* of ProMed4. (For simplicity in the present discussion we interchangeably refer to genes, and the RNAs and proteins that they code for. As we are dealing here with bacteria, this simplification is not too problematic, and BioBike knows how to automatically determine when translation is needed between these.) One way to address this biological question is to ask which proteins in ProMed4 have no ortholog—that is, no gene of similar apparent function (based upon sequence similarity)—in Pro9313. One can get an even finer bead on this question by examining microarray expression results for the genes that produce those proteins, asking which of those genes unique to ProMed4 demonstrate a significant light response (for example, are 2× up-regulated in a light stress experiment). Unfortunately, microarrays for the Prochlorococci have only recently been developed, so no such experimental work exists. However, there are a number of such studies using the related freshwater cyanobacterium: Synechocystis sp. strain PCC6803 (aka. s6803) [11]. Going one step further, one may focus specifically on the genes that are annotated as photosynthesis-related according to some formalization of gene function, such as the Gene Ontology (GO).

In sum, we can expand this question as follows:

Which photosynthesis-related proteins in ProMed4 have no ortholog in Pro9313 but do have an ortholog in s6803 such that the genes producing those proteins exhibits a light stress response (greater than 2× ratio in microarray data), and possibly are annotated as light-related genes?

One algorithm to find such genes might be:


For each photosynthesis-related gene/protein in ProMed4,
 Find its best protein-level ortholog
		  in Pro9313 and S6803,
When there is no Pro9313 ortholog,
  but there is a S6803 ortholog,
  and the expression ratio for the genes (mRNAs) that
    produce the S6803 ortholog are >2×
    up-regulated in the Hihara microarray dataset,
collect the ProMed4 gene/protein.


Expressed in the native BioBike language (BioLisp), this might be written as follows:


   (loop for pm4prot in (#^proteins promed4)
     as 9313ortho  =  (best-ortholog pm4prot pro9313)
     as 6803ortho  =  (best-ortholog pm4prot s6803)
   when (and (photosynthesis-related pm4prot)
        (null 9313ortho)
         6803ortho
        (> = (array-select 6803ortho Hihara1) 2.0))
     collect pm4prot)


Although this solution is concise and relatively efficient, its programming requires detailed knowledge of both the BioLisp programming language, and of how to call upon BioBike's knowledge resources. Moreover, this is a one-shot solution specific to this particular problem, not affording of significant reuse (i.e., methodological modularity). Nor does this approach get us any more than the solution reported as an opaque answer that cannot be unpacked into the method that solved it (provenance).

In contrast to this approach, the BioDeducta methodology allows us to more conveniently express and solve this problem while simultaneously offering methodological modularity and solution provenance. We begin by expressing our query in high-level terms familiar to the biologist, and then unpack each concept into modular conceptual constituents in the subject domain theory. It is the job of the theorem prover to figure out how to use the guidance offered by the subject domain theory to find a solution to the top query.

The query might be expressed as follows: What gene enables med4 to adapt to its light environment? Or, expressed in terms of a SNARK formal conjecture, does there exist a gene ?gene such that:

(adaptive-gene ?gene med4 light)?

The satisfaction of this conjecture in the subject domain theory is to be found by the theorem prover. Terms in the query preceded by question marks (?gene) are variables that will be plugged in by SNARK, if possible, in the process of providing a proof for the conjecture; in proving the existence of such entities, the theorem prover will be forced to find consistent values that fit these variables. These descriptions of genes and organisms that satisfy the conditions will then be extracted from the proof. The proof thus constitutes an explanation of why the extracted entities satisfy the desired conditions. The other terms (med4 and light) are constants that are fixed by the theory.

### Subject domain theory

The meanings of the symbols of a query, such as adaptive-gene, are defined by axioms of the subject domain theory. The theorem prover transforms the query in accordance with these axioms. Unlike a logic-programming system such as Prolog, the theorem prover is not constrained to process the query in the order given; rather it follows its own strategic controls.

The subject domain theory has three parts: (1) modular definitions that enable SNARK to translate the high-level query into a search procedure through subgoals that finally ground out in terms of ground knowledge and BioBike procedures, (2) simple ground knowledge that requires no internal (subgoal) computations, and (3) procedural attachments into the BioBike knowledge base that access computed knowledge and which may perform internal “hidden” computations. As we proceed with this exposition, it is important to keep in mind that these various resources are independent of the particular adaptive-gene query in the present example; rather, they are conceptually self-contained and modular in that they could be used in any number of queries regarding the properties of genes and proteins. (The subject domain theory will generally have been previously provided by either the knowledge base designers or by other biologists. Here, of course, we have written it for this problem, so it exhibits somewhat less generality. Elsewhere we could engage in a productive debate about how each axiom should properly be cast for maximum generality.)

In this presentation we begin from the goal, and work our way conceptually downward. Recall that we seek a proof of the theorem that establishes the existence of a gene (?gene) such that:

(adaptive-gene ?gene med4 light)

(More precisely we seek a refutation of the negation of this assertion. This process, called “proof by refutation”, is explained in detail in Russell and Norvig [12] ch. III.9.)

Here is the definition of what it means for a gene to be adaptive—that is, for it to be related to the way in which an organism adapts to its environment:


   (adaptive-gene ?gene1 ?organism1 ?dim)
     ⇔ [i.e., if and only if]
    (and
     (gene-in-organism ?gene1 ?organism1)
             (gene-semantics ?gene1 ?dim)
             (differentiating-gene ?gene1 ?organism1 ?dim)
             (differentially-regulated ?gene1 ?dim))


That asserts that a gene is related to the way in which an organism adapts to its environment along a particular environmental dimension (i.e., light, in the present example) if and only if four conditions are met: First, the gene must be a gene of the given organism. Second, the gene's putative function (per its explicit annotation, provided by the database) must be conceptually related to the relevant dimension. Third, the gene must (genomically) differentiate between organisms that live in environments that differ along the relevant dimension (here: light). And fourth, the gene must be differentially regulated in an experiment that explores the relevant dimension (again: light).

Importantly, none of the specifics of the query are explicit in this formulation of what it means to be an adaptive gene; this axiom is general to this sort of problem, and uses internal formulae, which are likewise general (and which we explain below). One could argue about whether this is precisely what one intends by the biological concept of “adaptive gene”, but it is easy to adjust this definition if one desires.

Continuing to unpack the meaning of terms: A differentiating gene (more precisely: a genomically differentiating gene) is one that exists in one organism and not in another that lives in a different niche, and where the niches differ along the relevant dimension:


   (differentiating-gene ?gene1 ?organism1 ?dim)
   ⇔
   (and
    (exists
     ((?organism2 :sort species))
     (and
      (differentially-ecotyped ?organism1 ?organism2 ?dim)
      (gene-in-organism ?gene1 ?organism1)
      (not (exists
                   ((?gene2 :sort gene))
                   (gene-has-ortholog-in-organism
                     ?gene1 ?gene2 ?organism2)
                   ))))


Note that this axiom asserts the existence of a second organism, unspecified in the formula that calls upon this axiom. When SNARK encounters the not exists clause it will attempt to find a gene (?gene2) that is an ortholog of the given gene (?gene1) in the second organism (?organism2). If there *is* such a gene, this branch of the search will *fail*, causing the specific gene ?gene1 to be rejected as a differentiating gene, in which case SNARK will try another. On the other hand, if there is no such gene, ?gene2, this branch of search will succeed, and the proof will continue. (Theorem-proving aficionados will note that this is true negation, not negation-as-failure; we need to know that no ortholog exists, not that we have merely failed to find one. There is a closed-world assumption applied to the procedural attachment: If the attachment finds no ortholog, we assert that no ortholog exists.)

We define the concept of being differentially ecotyped as follows: There is another organism (presumably in the same group as our target organism) that lives in a different environment regarding the specified dimension (light, in the present case). Here we very roughly approximate the qualities of light as low vs. high.


   (differentially-ecotyped ?organism1 ?organism2 ?dim)
   ⇔
   (or
    (and
     (organism ?organism :environment ?dim
                :quality high)
     (organism ?organism2 :environment ?dim
                :quality low))
    (and
     (organism ?organism :environment ?dim :quality low)
     (organism ?organism2 :environment ?dim
                :quality high)))


Note that this must be expressed in both directions (across the or) to handle the case in which ?organism1 is high and ?organism2 low, and vice versa. (Also, this will only work if the terms “High” and “Low” are both used by the author of the query and by the author of the subject domain theory. Any project of this sort will confront such terminological issues. We have chosen these particular terms because biologists refer to med4 as a the *high light strain* and mit9313 as a *low light strain* [10], so there is more likely to be agreement in this case.)

Finally, to determine differential regulation we use BioBike's knowledge of microarray experimental results on the given (light) dimension (i.e., from the Hihara et al. [11] data) to find a gene on a microarray in a relevant orthologous organism on which a relevant experiment has been conducted, and then to test for up-regulation of the ortholog:


   (differentially-regulated ?gene ?dim)
   ⇔
   (exists
    ((?experiment :sort experiment)
     (?organism3 :sort species)
     (?gene3 :sort gene))
   (and
    (experiment ?experiment :dimension ?dim
                  :organism ?organism3)
    (gene-has-ortholog-in-organism ?gene
                       ?gene3 ?organism3)
    (> (regulation-ratio ?gene3 ?experiment) 2.0)
    ))


Here regulation-ratio is a procedural attachment that returns a value from a given experiment for a given gene, and the whole (> ...) clause ensures that the gene under consideration has a significant (2× up-regulated) response in that experiment. (One may well wonder, after adding all these axioms, whether the subject domain theory is consistent. Asking SNARK to prove “false” will (eventually) root out inconsistencies. Unfortunately the time that this takes is unpredictable so one must decide when to stop it and accept that the theory is consistent.)

### Simple ground knowledge

In addition to the axiomatization above, and extensive knowledge of genes, organisms, ontologies, and microarray data built into BioBike and accessed by SNARK via procedural attachments (as described in the next section), we must provide knowledge that is specific to the present problem. Since the query is expressed in terms of the word “light”, most of this relates that term to various organisms and experiments.

These first three assertions tell SNARK that light varies along three qualitative dimensions, low, medium, and high, and that the light environments of the three organisms, mit9133, s6803, and promed4, are those niches respectively:


   (organism mit9313 :environment light :quality low)
   (organism s6803 :environment light :quality medium)
   (organism med4 :environment light :quality high)


These assertions are to be read as: The organism “mit9313” on the environmental dimension “light” has the quality value “low”, and so on. We must also relate the Hihara et al. [11] experiment to the light dimension, and indicate that this experiment was conducted on the Synechococcus sp. strain PCC6803 (s6803) organism:

(experiment hihara :dimension light :organism s6803)

If desired, an arbitrary amount of additional irrelevant knowledge could be added to make this example more realistic, but because theorem proving as used here is formally monotonic such additional knowledge would not change the results of the example, although it might slow down the proof process as false leads and dead ends are explored and rejected by the theorem prover.

### Procedural attachments

Finally, we provide concepts that are implemented by procedural attachment in the BioBike system:

- (gene-in-organism ?gene ?organism)—This relation asserts that ?gene is a gene of the given ?organism. Procedural attachments to this relation compute either the organism containing a given gene, or, inversely, all the genes contained in a given organism.
- (ortholog ?gene1 ?gene2)—BioBike has a built-in concept of two-way orthology between given genes (more precisely, between the proteins produced by given genes). A procedural attachment to this ortholog relation computes the (protein-based, two-way) gene orthologous to a given gene.
- (gene-has-ortholog-in-organism ?gene ?gene1 ?organism)

Given the concepts of gene-in-organism and ortholog, as above, it is easy to introduce an axiom that conceptually defines gene-has-ortholog-in-organism as follows:


   (gene-has-ortholog-in-organism ?gene1 ?gene2 ?organism)
    ⇔
     (and
       (ortholog ?gene1 ?gene2)
       (gene-in-organism ?gene2 ?organism))


That is, a given gene has an ortholog in a specific given organism if and only if there is a gene (?gene2) in the target organism and that gene is orthologous (as defined above) to our given gene.

When SNARK encounters expressions with procedural attachments, such as:

(gene-in-organism ?gene med4)

a data source is invoked that yields all the genes in the given organism (Procholorococcus sp. strain Med4). In different branches of the search space, the variable ?gene will be systematically replaced by each of these genes, respectively. The procedural-attachment mechanism allows the theorem prover to behave as if the axioms of the theory express the complete list of the genes of promed4, while in reality that knowledge is imported from the external data source only when it is needed.

Two somewhat more problem-specific primitives are needed in order to work with microarray data, and to examine gene annotations:

- (regulation-ratio ?gene3 ?experiment)—This function returns a number representing the mean up-or-down regulation ratio of a given gene in a given microarray experiment. The experiment name and gene object must both be given. In the present example the data from the experiment of Hihara et al. [11] is available and has been massaged as described in Appendix S1 to provide a mean regulation ratio for each gene.
- (photosynthesis-related ?gene)—Finally, among the primitives that we take as built in via BioBike procedural attachments is a test for a gene object being photosynthetically related. This test could work in any number of ways, ranging from reading the annotation field for the gene, to reading information from a functional ontology regarding the given gene, to asking the biologist's opinion. How this is implemented is not relevant to the present discussion so we have simply implemented it to search the gene's Cyanobase (www.kazusa.or.jp/cyano/) annotations (which are built into the BioBike knowledge base) for the string “light” or “photo”. This is not a highly certain way of finding photosynthetically related genes (esp. given the concerns raised by Shrager [13]), but it will do for the present example.

We also add assertions that tell us that when we ask about the light semantics along the light dimension, SNARK should make use of this built-in photosynthesis-related predicate to determine whether or not this is the case for a given gene:


   (gene-semantics ?gene light)
   ⇔
   (photosynthesis-related ?gene)


We will return in the discussion to consider other interesting ways in which this axiom could be expressed.

## Results

We provided BioDeducta all of the above and asked it to find a gene (and other related terms) that satisfy our query:

(adaptive-gene ?gene med4 light)

Once the proof is complete, the theorem prover extracts an answer to the query by examining what term replaces the variables ?gene: PMED4.PMM0817. This is the only answer for this specific query, and it is correct. This proof takes about a minute to find this answer (on a dual Athlon Linux processor with 1G memory). By examining the proof (provided in the online supplementary materials) we find that in order to obtain this answer, SNARK discovered these auxiliary answers, which can be understood by finding their locations in the axioms of the subject domain theory, above (and Appendix S2):


   ?gene: #$PMED4.PMM0817
   ?organism2: #$prochlorococcus_marinus_mit9313
   ?experiment: HIHARA  ?organism3: #$synechocystis_pcc6803
   ?gene3: #$S6803.ssr2595


In other words, a low-light organism that has no ortholog to ?gene is prochlorococcus_marinus_mit9313 (pro9313). Experiments were performed by Hihara on the organism synechocystis_pcc6803 (s6803), and a high regulation ratio was discovered in those experiments on gene S6803.ssr2595, which is an ortholog of PMM0817. The annotation for PMM0817 reads: “possible high-light inducible protein”. Indeed, the high-light inducible (hli) proteins (genes) have been previously identified as being possibly involved in the adaptation of the Procholorococci to different environmental niches by Bhaya et al. [10], who identified members of the hli gene family in seven cyanobacterial genomes, including those of Prochlorococcus sp. strain Med4 nad Prochlorococcus sp. strain MIT9313. PMM0817 is called “hli17” in Bhaya et al [10].

We conducted a number of additional experiments, demonstrating that BioDeducta's results mirror the results computed by the equivalent BioLisp programs run in the same BioBike database. For example, as was mentioned above, one may quibble with the specifics of our choice of definitions, but it is easy to change the meanings of terms, or to add alternative formulations that modularly work together with existing axioms. For example, one might wish to change the definition of adaptive-gene as follows:


   (adaptive-gene ?gene1 ?organism1 ?dim)
   ⇔
   (and
    (gene-in-organism ?gene1 ?organism1)
    (differentiating-gene ?gene1 ?organism1
    			   ?organism2 ?dim))


Note that the gene-semantics and differentially-regulated clauses are deleted. Re-executing the proof with this axiom produces 340 results (for ?organism2  =  pro9313). These (and other variations of this example) were checked by equivalent BioLisp code.

Having demonstrated how a combination of axiomatic reasoning, answer extraction, and procedural attachment may offer biologists access to powerful biocomputing analyses, we next turn to discussion of some closely related work, followed by discussion of some of the issues and opportunities raised by our work.

## Discussion

### Closely related work

A bevy of activity in biocomputing is concerned with the formal representation and reasoning about biological pathways. An excellent example of this is the Pathway Logic work based on the Maude rewriting logic paradigm [14], [15]. Although based upon somewhat different technology (rewriting systems vs. first-order theorem proving), we share the methodology of expressing a formal subject-domain theory, and then reasoning from it to transform a conjecture into a form that affords various sorts of analyses, such as model checking.

The fact that Pathway Logic operates in the domain of biological signaling pathways, whereas the examples in this paper are in the domain of genomic conjectures, is an incidental difference resulting from our respective choices of problems. Because we both use explicit models, we can in principle do one another's problems by representing one another's subject domain theories. That is, given a subject domain theory containing axioms that define temporally related biological events, BioDeducta will do exactly the same work as Pathway Logic. Indeed, in the BioBike Live Tutorials that come with the BioBike system we develop several other examples, including one that analyzes protein regulation models (see the Software Availability section, below). The subject domain theory for that example includes axioms such as:


   (and (controls ?protein1 ?protein2)
      (controls ?protein2 ?protein3))
   ⇒ [i.e., implies]
   (controls ?protein1 ?protein3))


One can consider Maude as a sub-logic of SNARK, specialized to certain forms of rewriting-based reasoning. Although far less general than SNARK, Maude is very efficient for certain kinds of problems, such as reachability (e.g., Can a certain molecule be generated from given precursors?) What is ultimately needed is an integration of SNARK with Maude (and other tools) so that queries that are solvable in principle in first-order logic can be solved efficiently with specialized logically sound algorithms such as the model-checking techniques available in Maude. (We thank Mark-Oliver Stehr for this insightful discussion.)

Some other systems, such as HyBrow [16], try to decide the validity of a declarative conjecture from biological knowledge and/or data. The user of HyBrow expresses fully grounded hypotheses (i.e., conjectures without variables) as sets of temporally related biological events. HyBrow evaluates these hypotheses against genomic and microarray data and may offer alternative “neighboring” hypotheses with improved fit to the data. Although hypotheses in HyBrow are fully grounded, whereas the BioDeducta example given in the present paper contains variables, this is an illusory difference; we could just as well have grounded the adaptive-gene conjecture with specific genes, in which case the BioDeducta methodology looks very similar to HyBrow's. Indeed, grounding the conjecture in specific genes (and other abstracted variables) is precisely how SNARK goes about proving it. Moreover, HyBrow's ability to propose “improved” hypotheses is orthogonal to its conjecture validation mechanism, and is a property of many other systems (e.g., [17]). Moreover, the HyBrow concept of “neighboring” hypotheses is ad hoc and dependent upon the specific representation of conjectures, which is (as described above) built into HyBrow's code and is not explicit. Thus, although BioDeducta's ability to replace variables seems to be narrower than HyBrow's neighboring hypothesis concept, this is illusory because of the choice of representation in the present examples. If we had used an example that looked like a HyBrow temporal model, and provided an axiomatization for it, the BioDeducta representation could afford the same representational “hinges” that HyBrow uses to compute neighboring hypotheses. Moreover, because of the declarative axiomatization of the subject domain theory in BioDeducta, these representational hinges could be manipulated much more directly than is the case in HyBrow, which is a “black box” program. In these ways BioDeducta is more general than HyBrow.

Furthermore, as with Pathway Logic, the apparent difference in domain between Hybrow and BioDeducta is merely a happenstance of the examples we have chosen. Axioms such as:


    (precedes ?event1 ?event2)
     □
    (exists ((?time1b :sort time)
       ...etc...)
   (and
     (event-begins-at ?event1 ?time1b)
     (event-ends-at ?event1 ?time1e)
     (event-begins-at ?event2 ?time2b)
     (event-ends-at ?event1 ?time2e)
     (non-overlapping-ranges ?time1b ?time1e
                   ?time1b ?time1e)))


could serve the purpose in BioDeducta of computing the same sorts of analyses as HyBrow. Indeed, SNARK has as a built-in version of the Allen temporal calculus [18] for reasoning about time points and intervals in just this manner, so building this logic would not be difficult.

The central difference between BioDeducta (or Pathway Logic) and HyBrow is that BioDeducta is an inference engine armed with an explicit axiomatization of its subject domain theory, whereas HyBrow is an ad hoc program in which the equivalent of the subject domain theory is built into special-purpose code. Therefore, HyBrow cannot carry out inference and so HyBrow users cannot use high-level descriptions that are grounded by a subject domain theory in a principled way. Furthermore, HyBrow cannot give explanations that involve such transformations (even if they were carried out in the ad hoc HyBrow code). In these important ways, BioDeducta goes well beyond HyBrow and HyBrow-like systems.

### Issue: Efficiency and the undecidability of full first-order logic

Being very general, SNARK's proof process will usually be slower than specially written BioLisp programs because the latter may take advantage of specific properties of the problem to speed up search. Our solution to the light acclimation query is slower than a cleverly crafted program, but faster than a naive one. It does, however, require theory-specific domain engineering and strategic work to achieve good performance. This may be chalked up to the price one pays for the flexibility afforded by using a full first-order theorem prover with an explicit subject domain theory.

Full first-order logic is undecideable in general, meaning that there is no hope of developing a fast *general-purpose* reasoning capability for it. However, within a particular subject-domain theory it is often possible to develop strategic controls that allow the theorem prover to exhibit performance rivaling that of special-purpose systems. Moreover, where the objects involved are finite, as is the case here for genomes, etc., it is likely that most theories are naturally decidable (although one can certainly go out of one's way to build an undecideable theory if one wishes to do so).

Through the use of weights and clause ordering, the designer of the subject domain theory can force certain sub-formulae to be treated before others. In the present example, for instance, some symbols have procedural attachments while others do not. Some procedural attachments, such as gene-in-organism, can be very expensive, since an organism may have thousands of genes, whereas others, such as gene-has-ortholog-in-organism, are relatively cheap because a gene usually has only a small number of orthologs in a given organism. We can weight symbols in proportion to the expected number of solutions offered by the corresponding attached procedure.

Useful properties may also be stated for relations, such as symmetry or reflexivity. For example, ortholog can be declared to be commutative (i.e., symmetric) but in some cases this gives poorer running time. One can also state a reflexivity axiom: (ortholog ?gene ?gene), which may will allow some clauses to be dropped by subsumption and improve the search efficiency. Not only is the present theory decidable, but by judicious use of these mechanisms it is also efficient.

Regardless of all these manipulations, given that s6803 has 3722 genes, promed4 has 1760, and pro9313 has 2328, even if it takes several minutes for SNARK to compute a solution, this is far less time than it would take a biologist to do the same work manually or using spreadsheets.

### Issue: The complexity of domain theory formulation

Even aside from these tuning details, the mere construction of a subject domain theory is a complex and error-prone task. Fortunately, in theory it need be done only once for each subject domain. Furthermore, we do not need to begin from scratch but can import appropriate sections of subject domain theory from such standards as Cycorp's OpenCyc [19] and Teknowledge's SUMO [20], [21] and more specific ontologies for the selected subject domain such as the axiomatization of molecular biology included in the Library of Ontologies of the Laboratory for Applied Ontology [http://www.loa-cnr.it/], which has been under development for many years. Other ontologies that are being developed for biology include the Ontolingua Molecular Biology Theory (www.loa-cnr.it/medicine/molecular-biology) and the Open Biological Ontologies project (obo.sourceforge.net/main.html); see also [22].

One can also build a subject domain theory by composing simpler theories. One should think of the subject domain theory as a sort of dictionary of biological concepts; it modularly describes how concepts are cached out in terms of other (simpler) concepts, eventually reaching ground facts or underlying computations. The development of any dictionary is not trivial, but because of the generality of the theorem prover, the in-principle modularity of the subject domain theory is essentially guaranteed (although not necessarily its efficiency, unless steps such as those described above are taken as well).

Of course, merging subject domain theory components may not be straightforward; different theories may use different symbols for describing the same concept, or may use the same symbol with different meanings. The notions of theory morphism and colimit, obtained from the mathematical theory of categories [23], has been found to be valuable for this purpose [24], [25]. A theory morphism is a meaning-preserving mapping that identifies symbols in one theory with other symbols in a different theory; the colimit then allows us to compose the theories, taking these identifications into account. In this way, one can combine theories even if component theories use different vocabulary for the same concept, or the same vocabulary for different concepts. Specware [26] is a category-theory-based framework that implements essential theory-manipulation operations, including theory morphism and colimit, required for the composition of multiple ontologies and theories. It provides an interface that allows us to access and generate SNARK theories and proofs.

Regardless of the specific approaches to mitigating issues of efficiency and of the completeness and correctness of the subject domain theory (not to mention ambiguity and arguments about definitions!), problems are bound to arise in any project of the sort we have described. As with any such project, only the long-term efforts of a dedicated community can work these out. We offer technology that is powerful enough to afford correct, complete, efficient, and explicit solutions—the biocomputation community will, over time, work out the details. BioBike is a collaborative platform within which such a community can engage in efforts of this sort.

### Opportunities: Quasi-natural language and query elicitation

We opened this paper with the biologist in increasing need of the ability to conduct novel computational analyses without programming, and offered BioDeducta as an approach to this. Whereas BioDeducta may provide significant opportunities in terms of methodological modularity and provenance, it may still be difficult to imagine biologists expressing queries in SNARK's logical notation (or, similarly, writing SNARK axioms). One approach to this problem is to provide graphical support for query formulation, such as was done in NASA's Amphion system [27], [28] which automatically produces software for planetary astronomers. Amphion accepts user queries formulated with the help of a graphical query-formulation guide, producing a diagrammatic representation of the query. The diagram is then translated into a logical conjecture, which is passed to SNARK for proof.

Another approach to simplifying the query (or theory) formulation task is to use quasi-natural-language. Whereas true natural language programming (or at least querying) has been a holy grail of AI since time immemorial, we do not imagine that this is possible in the near term, even in narrow domains such as biocomputing. However, the fact that the BioDeducta subject domain theory is explicitly formulated makes certain sorts of quasi-natural language a real possibility. SRI's GeoLogica and QUARK [29] are both experimental systems that use deductive methods very similar to the way this is done in BioDeducta to compose heterogeneous data and software components: A logical form is presented as a conjecture to SNARK, and an answer to the query is extracted from the proof. While GeoLogica responds to queries posed by an Earth systems scientist, QUARK serves as an assistant to intelligence analysts. Both systems accept queries in English which are translated into logical form by a natural-language parser.

We have conducted preliminary experiments with natural language in BioDeducta, using the same method as described for the GeoLogica system [29]. Briefly, a query is parsed by Gemini, a general parser, and translated into a logical form, phrased as a conjecture, and submitted to SNARK. As above, the proof produces a set of bindings for free variables in the logical form produced by Gemini. These bindings constitute answers to the question.

For example, the query

“Find a gene that pertains to Promed4 and that does not have an ortholog in Pro9313.”

translates to a logical form that is thence translated by a language subject domain theory into a BioDeducta conjecture, which is thence proved, just as was done in the hli example, above. In the end, the proof produces the answer

#$PMED4.PMM0030

Examining alternative proofs to the theorem produces multiple answers:

#$PMED4.PMM0038

#$PMED4.PMM0051

This example required 5 seconds to produce the first answer, and additional answers were almost instantaneous.

Many other sorts of queries can be addressed by BioDeducta using this approach. Examples include

“Does pmm0226 not have an ortholog in mit9313?”

“What is the Hihara mean regulation ratio of pmm0226?”

Moreover, various semantically equivalent forms are acceptable. For example, one can ask about the “hihara ratio” or the “hihara regulation ratio”, which are taken to be synonymous.

Much as this is encouraging, we do not pretend to have solved the natural language problem for biocomputing. To express such queries (in any language, natural or otherwise) users must know a great deal about the system's capabilities. Natural language provides the illusion that the system can understand everything whereas it is difficult for a system to engage the user in a natural language dialogue to indicate what it does and does not understand.

While naïve users may not be able to formulate the appropriate logical query, we may be able to guide such users to formulate logical queries even if they are ignorant of logical notation or the vocabulary of the subject domain theory. This approach depends upon the use of a sorted theory, one in which each constant is assigned a sort, that is an indicator of a class to which it belongs; thus, promed4 may be declared to be of sort organism (or sort bacterium, a subsort of organism). Similarly, each function symbol is given a declaration of the sorts of arguments it requires and the sort of value it produces, and each relation has a declaration of the sorts of arguments it expects. Such declarations are valuable for a theorem prover in that they restrict search, admit shorter proofs, allow some error detection, and permit more concise axioms and queries. But a sorted theory is of special value in that it allows query elicitation. Let us imagine that a user was trying to formulate the complex query discussed above. The user might select the term promed4 from a menu of known organisms. Since promed4 is of sort organism, the system would offer a menu of relations and functions that accept terms of sort organism as arguments including, for example, adaptive-gene and gene-in-organism. An English paraphrase of the meanings of these relations could be provided. Alternatively, the user could type an approximation to the desired operator, and the system might offer near matches that accept terms of appropriate sort as arguments. As expression formation proceeds, the types of functions and relations constrain the types of arguments, and vice versa until the query is as complete as the user can make it, at which point it is turned over to SNARK. This same mechanism might be used to introduce new content or axioms into the system, thus enabling a subject domain expert who is ignorant of the language of logic or of the vocabulary of the existing subject-domain theory to be guided to add new axioms to the theory.

### Conclusions: On proof and provenance

The goal of BioDeducta is to put biological computation directly into the hands of biologists themselves—to enable them to manipulate biological knowledge and data in an interactive computational environment, and to produce results that are backed up by explicit explanations (the proofs). But biocomputation is not a simple art, often requiring one to program complex navigations within and between complex knowledge bases. Although BioBike offers the full power of a mature programming language, it puts the burden of figuring out how to navigate the knowledge bases entirely upon the biologist/users themselves. BioDeducta can in principle assist the user by taking advantage of the guidance of an explicit subject domain theory to find its way through the knowledge base to answer complex queries. Of course, the more meta-knowledge is available to describe knowledge bases and their relationships, the more complete is BioDeducta's ability to offer assistance in this regard.

In concluding, we wish to emphasize an aspect of the present approach that helps address a critical problem in computational biology: the problem of provenance—that is, tracking how results are calculated, especially as it applies in the annotation of biological function.

The concept of gene function is highly problematic for a number of reasons, not the least of which being that the way in which function is determined by the annotator is not generally made explicit in the annotation. As more and more genomes come online faster and faster, functional annotation is more and more being done through computational methods rather than by experiment. Because the provenance—the data behind such annotations—is not stored, there is the potential—in fact, the near certainty!—of propagating errors and, complementarily, failing to propagate corrections [13]. One approach to this is to record such provenance, but often there is nothing worthy of recoding because the annotation results from an opaque program such as HyBrow [16]. The general BioDeducta method offers an approach to this problem, as follows.

Recall that the explicit subject domain theory is not so much a theory of biology as a description of the way to expand high-level biological concepts in terms of more primitive concepts, finally reaching “ground” terms and functions in the knowledge base or BioBike functions. By virtue of this, the proof constructed by SNARK in the process of proving a given conjecture forms an explicit trace or “explanation”, including the specific axioms used, and the ways in which the variables were bound in the axioms that lead to a given result; the more explicit the subject domain theory, the more detailed the explanation. By virtue of this fact, BioDeducta proofs represent precisely the sort of explicit provenance whose absence is endangering the very underpinnings of molecular biology, and by recoding the proofs along with the results that underpinning could be, at least in part, restored!

### Software and example availability

BioDeducta is SNARK+BioBike. SNARK is built-in to the BioBike demo server, accessible through www.biobike.org, and is usable without having to register. Because of a time limit on computations taking place in the demo server only simple BioDeducta proofs can be accomplished on that server. Users wishing to seriously experiment with BioDeducta should either ask Jeff Shrager (jshrager@stanford.edu) for an account on a nondemo BioBike server, or should install BioBike and SNARK themselves. Both of these are Common Lisp programs and are open source freeware for research applications. The BioBike installation instructions (also at www.biobike.org) explain how to download and install SNARK as well.

The examples in this paper are available as BioBike Live Tutorials, also at www.biobike.org. The BioBike Live Tutorial system walks students through the entire process of developing and running the examples, and includes explanation of some of the more important SNARK parameters.

## Supporting Information

Appendix S1 Preparation of the Hihara et al. (2001) Data
(0.03 MB DOC)

Appendix S2 Complete Refutation Proof for the adaptive gene conjecture resulting in PMM0817
(0.06 MB DOC)
