# Supplementary material for: Deductive Biocomputing
Source: PLoS One. 2007 Apr 4;2(4):e339. doi: 10.1371/journal.pone.0000339 (PMC1838522; doi:10.1371/journal.pone.0000339)
Supplement: Appendix S1 — Preparation of the Hihara et al. (2001) Data (0.03 MB DOC) [file pone.0000339.s001.doc]

# Appendix 1. Preparation of the Hihara et al. (2001) Data

We downloaded the raw data for the Hihara et al. (2001) experiment from the KEGG Expression Database (http://www.genome.jp/kegg/expression/). The data arrives in 20 separate files: 6 at 15 min., 6 at 1 hr., 4 at 6 hrs., and 4 at 15 hrs. exposure to high light, each containing 3079 rows of data (one gene per row). The data in each file was independently (by file) normalized in accord with the method of Hihara et al., and ratios computed for “target” channels against each “control” channel. Next the mean of all the time points for each gene was independently (by gene) computed from the normalized data across all the files. Before BioDeducta experiments are run, a table is preloaded into BioBike that enables scalar-time lookup of these mean values given a BioBike gene object. If the given gene did not appear on the microarray (or the ratio could not be calculated), it will not appear in this table. In this case a zero (0.0) is returned for its regulation ratio. It could be argued that an unlocatable gene should return a ratio of 1.0 (neither up nor down regulated) or some sort of error indication to reject it from the search. However, in the present example we are searching only for genes with ratio > 2.0, so whatever is returned, so long as it is not >2.0, will cause an unlocatable gene to be rejected.
