# Supplementary material for: Deductive Biocomputing
Source: PLoS One. 2007 Apr 4;2(4):e339. doi: 10.1371/journal.pone.0000339 (PMC1838522; doi:10.1371/journal.pone.0000339)
Supplement: Appendix S2 — Complete Refutation Proof for the adaptive gene conjecture resulting in PMM0817 (0.06 MB DOC) [file pone.0000339.s002.doc]

# Appendix 2. Complete Refutation Proof for the adaptive gene conjecture resulting in PMM0817:

07/20/06 15:01:35 :

::

; Loading /home/jshrager/snark/elhai2.lisp

; Running SNARK from /usr/local/biotools/snark/current/snark-system.lisp in Allegro CL Enterprise Edition 8.0 [Linux (x86)] (Mar 18, 2006 15:45) on #x7f0100 at 2006-07-20T15:00:40

Warning: - is being used as a 1-ary function; it is also a 2-ary function.

Warning: / is being used as a 1-ary function; it is also a 2-ary function.

; The current SNARK option values are

; (USE-RESOLUTION T)

; (USE-HYPERRESOLUTION NIL)

; (USE-NEGATIVE-HYPERRESOLUTION NIL)

; (USE-UR-RESOLUTION NIL)

; (USE-UR-PTTP NIL)

; (USE-PARAMODULATION NIL)

; (USE-FACTORING T)

; (USE-EQUALITY-FACTORING NIL)

; (USE-CONDENSING T)

; (USE-RESOLVE-CODE NIL)

; (USE-CODE-FOR-EQUALITY T)

; (USE-UNIT-RESTRICTION NIL)

; (USE-INPUT-RESTRICTION NIL)

; (USE-LITERAL-ORDERING-WITH-RESOLUTION LITERAL-ORDERING-A)

; (USE-LITERAL-ORDERING-WITH-HYPERRESOLUTION NIL)

; (USE-LITERAL-ORDERING-WITH-NEGATIVE-HYPERRESOLUTION NIL)

; (USE-LITERAL-ORDERING-WITH-UR-RESOLUTION NIL)

; (USE-LITERAL-ORDERING-WITH-PARAMODULATION LITERAL-ORDERING-A)

; (USE-SUBSUMPTION T)

; (USE-SUBSUMPTION-BY-FALSE :FALSE)

; (USE-SIMPLIFICATION-BY-UNITS T)

; (USE-SIMPLIFICATION-BY-EQUALITIES NIL)

; (USE-TERM-ORDERING :RPO)

; (USE-DEFAULT-ORDERING NIL)

; (ORDERING-FUNCTIONS>CONSTANTS T)

; (RPO-STATUS :LEFT-TO-RIGHT)

; (KBO-STATUS :LEFT-TO-RIGHT)

; (KBO-VARIABLE-WEIGHT 1)

; (USE-INDEFINITE-ANSWERS NIL)

; (USE-CONDITIONAL-ANSWER-CREATION NIL)

; (USE-ANSWERS-DURING-SUBSUMPTION T)

; (USE-CONSTRAINT-SOLVER-IN-SUBSUMPTION NIL)

; (ALLOW-SKOLEM-SYMBOLS-IN-ANSWERS T)

; (REWRITE-ANSWERS NIL)

; (REWRITE-CONSTRAINTS NIL)

; (USE-FUNCTION-CREATION NIL)

; (USE-REPLACEMENT-RESOLUTION-WITH-X=X NIL)

; (USE-PARAMODULATION-ONLY-INTO-UNITS NIL)

; (USE-PARAMODULATION-ONLY-FROM-UNITS NIL)

; (USE-SINGLE-REPLACEMENT-PARAMODULATION NIL)

; (ASSERT-CONTEXT :ROOT)

; (ASSERT-SUPPORTED NIL)

; (ASSUME-SUPPORTED T)

; (PROVE-SUPPORTED T)

; (ASSERT-SEQUENTIAL NIL)

; (ASSUME-SEQUENTIAL NIL)

; (PROVE-SEQUENTIAL NIL)

; (NUMBER-OF-GIVEN-ROWS-LIMIT NIL)

; (NUMBER-OF-ROWS-LIMIT NIL)

; (AGENDA-LENGTH-BEFORE-SIMPLIFICATION-LIMIT 10000)

; (AGENDA-LENGTH-LIMIT 10000)

; (RUN-TIME-LIMIT 100)

; (ROW-WEIGHT-LIMIT NIL)

; (ROW-WEIGHT-BEFORE-SIMPLIFICATION-LIMIT NIL)

; (LEVEL-PREF-FOR-GIVING NIL)

; (VARIABLE-WEIGHT 1)

; (AGENDA-ORDERING-FUNCTION ROW-WEIGHT+DEPTH)

; (PRUNING-TESTS (ROW-WEIGHT-LIMIT-EXCEEDED))

; (PRUNING-TESTS-BEFORE-SIMPLIFICATION (ROW-WEIGHT-BEFORE-SIMPLIFICATION-LIMIT-EXCEEDED))

; (USE-CLAUSIFICATION T)

; (USE-EQUALITY-ELIMINATION NIL)

; (USE-MAGIC-TRANSFORMATION NIL)

; (USE-AND-SPLITTING NIL)

; (USE-AC-CONNECTIVES T)

; (USE-KIF-REWRITES NIL)

; (USE-LISP-TYPES-AS-SORTS T)

; (USE-NUMBERS-AS-CONSTRUCTORS T)

; (USE-CODE-FOR-NUMBERS T)

(Refutation

(Row definition-of-gene-in-organism-code

(or (gene-in-organism ?gene ?organism)

(not (gene-in-organism-code ?gene ?organism)))

assertion)

(Row 7

(organism |hashdollar-prochlorococcus_marinus_mit9313| :environment

light :quality low)

assertion)

(Row 8

(organism |hashdollar-prochlorococcus_marinus_med4| :environment

light :quality high)

assertion)

(Row 9

(experiment hihara :dimension light :organism

|hashdollar-synechocystis_pcc6803|)

assertion)

(Row defn-of-adapted-gene

(or (adaptive-gene ?gene ?organism ?x)

(not (gene-in-organism ?gene ?organism))

(not (gene-semantics ?gene ?x))

(not (differentiating-gene ?gene ?organism ?x))

(not (differentially-regulated ?gene ?x)))

assertion)

(Row definition-of-differentiating-gene

(or (differentiating-gene ?gene ?organism ?x)

(not (differentially-ecotyped ?organism ?species ?x))

(not (gene-in-organism ?gene ?organism))

(gene-has-ortholog-in-organism ?gene

(snark-user::gene-skolemmqlk2 ?gene ?species) ?species))

assertion)

(Row defn-of-differentially-regulated

(or (differentially-regulated ?gene ?x)

(not (experiment ?experiment :dimension ?x :organism ?species))

(not (gene-has-ortholog-in-organism ?gene ?gene1 ?species))

(not (> (regulation-ratio ?gene1 ?experiment) 2.0)))

assertion

Answer (defn-of-differentially-regulated ?gene

?x ?gene1 ?species))

(Row defn-of-differentially-ecotyped

(or (differentially-ecotyped ?organism ?organism1 ?x)

(not (organism ?organism :environment ?x :quality high))

(not (organism ?organism1 :environment ?x :quality low)))

assertion)

(Row defn-of-gene-semantics-light

(or (gene-semantics ?gene light)

(not (photosynthesis-related ?gene)))

assertion)

(Row 31

(not

(adaptive-gene ?gene |hashdollar-prochlorococcus_marinus_med4|

light))

negated_conjecture

Answer (snark::answer-- (list ?gene)))

(Row 32

(or

(not

(gene-in-organism ?gene

|hashdollar-prochlorococcus_marinus_med4|))

(not (gene-semantics ?gene light))

(not

(differentiating-gene ?gene

|hashdollar-prochlorococcus_marinus_med4| light))

(not (differentially-regulated ?gene light)))

(resolve 31 defn-of-adapted-gene)

Answer (snark::answer-- (list ?gene)))

(Row 34

(or

(not

(gene-in-organism-code ?gene

|hashdollar-prochlorococcus_marinus_med4|))

(not (gene-semantics ?gene light))

(not

(differentiating-gene ?gene

|hashdollar-prochlorococcus_marinus_med4| light))

(not (differentially-regulated ?gene light)))

(resolve 32 definition-of-gene-in-organism-code)

Answer (snark::answer-- (list ?gene)))

(Row 872

(or (not (gene-semantics |hashdollar-PMED4.PMM0817| light))

(not

(differentiating-gene |hashdollar-PMED4.PMM0817|

|hashdollar-prochlorococcus_marinus_med4| light))

(not (differentially-regulated |hashdollar-PMED4.PMM0817| light)))

(resolve 34 :code-for-gene-in-organism-code)

Answer (snark::answer-- (list |hashdollar-PMED4.PMM0817|)))

(Row 3468

(or

(not

(differentiating-gene |hashdollar-PMED4.PMM0817|

|hashdollar-prochlorococcus_marinus_med4| light))

(not (differentially-regulated |hashdollar-PMED4.PMM0817| light)))

(rewrite (resolve 872 defn-of-gene-semantics-light) :code-for-photosynthesis-related)

Answer (snark::answer-- (list |hashdollar-PMED4.PMM0817|)))

(Row 3469

(or

(not (experiment ?experiment :dimension light :organism ?species))

(not

(gene-has-ortholog-in-organism |hashdollar-PMED4.PMM0817| ?gene

?species))

(not (> (regulation-ratio ?gene ?experiment) 2.0))

(not

(differentiating-gene |hashdollar-PMED4.PMM0817|

|hashdollar-prochlorococcus_marinus_med4| light)))

(resolve 3468 defn-of-differentially-regulated)

Answer (or (snark::answer-- (list |hashdollar-PMED4.PMM0817|))

(defn-of-differentially-regulated

|hashdollar-PMED4.PMM0817| light ?gene ?species)))

(Row 5409

(or

(not

(gene-has-ortholog-in-organism |hashdollar-PMED4.PMM0817| ?gene

|hashdollar-synechocystis_pcc6803|))

(not (> (regulation-ratio ?gene hihara) 2.0))

(not

(differentiating-gene |hashdollar-PMED4.PMM0817|

|hashdollar-prochlorococcus_marinus_med4| light)))

(resolve 3469 9)

Answer (or (snark::answer-- (list |hashdollar-PMED4.PMM0817|))

(defn-of-differentially-regulated

|hashdollar-PMED4.PMM0817| light ?gene

|hashdollar-synechocystis_pcc6803|)))

(Row 5411

(not

(differentiating-gene |hashdollar-PMED4.PMM0817|

|hashdollar-prochlorococcus_marinus_med4| light))

(rewrite (resolve 5409 :code-for-gene-has-ortholog-in-organism) :code-for-regulation-ratio :code-for->)

Answer (or (snark::answer-- (list |hashdollar-PMED4.PMM0817|))

(defn-of-differentially-regulated

|hashdollar-PMED4.PMM0817| light

|hashdollar-S6803.ssr2595|

|hashdollar-synechocystis_pcc6803|)))

(Row 5413

(or

(not

(differentially-ecotyped |hashdollar-prochlorococcus_marinus_med4|

?species light))

(not

(gene-in-organism |hashdollar-PMED4.PMM0817|

|hashdollar-prochlorococcus_marinus_med4|))

(gene-has-ortholog-in-organism |hashdollar-PMED4.PMM0817|

(snark-user::gene-skolemmqlk2 |hashdollar-PMED4.PMM0817| ?species)

?species))

(resolve 5411 definition-of-differentiating-gene)

Answer (or (snark::answer-- (list |hashdollar-PMED4.PMM0817|))

(defn-of-differentially-regulated

|hashdollar-PMED4.PMM0817| light

|hashdollar-S6803.ssr2595|

|hashdollar-synechocystis_pcc6803|)))

(Row 5416

(or (not (organism ?species :environment light :quality low))

(not

(gene-in-organism |hashdollar-PMED4.PMM0817|

|hashdollar-prochlorococcus_marinus_med4|))

(gene-has-ortholog-in-organism |hashdollar-PMED4.PMM0817|

(snark-user::gene-skolemmqlk2 |hashdollar-PMED4.PMM0817| ?species)

?species))

(rewrite (resolve 5413 defn-of-differentially-ecotyped) 8)

Answer (or (snark::answer-- (list |hashdollar-PMED4.PMM0817|))

(defn-of-differentially-regulated

|hashdollar-PMED4.PMM0817| light

|hashdollar-S6803.ssr2595|

|hashdollar-synechocystis_pcc6803|)))

(Row 5422

(or

(not

(gene-in-organism |hashdollar-PMED4.PMM0817|

|hashdollar-prochlorococcus_marinus_med4|))

(gene-has-ortholog-in-organism |hashdollar-PMED4.PMM0817|

(snark-user::gene-skolemmqlk2 |hashdollar-PMED4.PMM0817|

|hashdollar-prochlorococcus_marinus_mit9313|)

|hashdollar-prochlorococcus_marinus_mit9313|))

(resolve 5416 7)

Answer (or (snark::answer-- (list |hashdollar-PMED4.PMM0817|))

(defn-of-differentially-regulated

|hashdollar-PMED4.PMM0817| light

|hashdollar-S6803.ssr2595|

|hashdollar-synechocystis_pcc6803|)))

(Row 5425

(or

(not

(gene-in-organism-code |hashdollar-PMED4.PMM0817|

|hashdollar-prochlorococcus_marinus_med4|))

(gene-has-ortholog-in-organism |hashdollar-PMED4.PMM0817|

(snark-user::gene-skolemmqlk2 |hashdollar-PMED4.PMM0817|

|hashdollar-prochlorococcus_marinus_mit9313|)

|hashdollar-prochlorococcus_marinus_mit9313|))

(resolve 5422 definition-of-gene-in-organism-code)

Answer (or (snark::answer-- (list |hashdollar-PMED4.PMM0817|))

(defn-of-differentially-regulated

|hashdollar-PMED4.PMM0817| light

|hashdollar-S6803.ssr2595|

|hashdollar-synechocystis_pcc6803|)))

(Row 5427

(gene-has-ortholog-in-organism |hashdollar-PMED4.PMM0817|

(snark-user::gene-skolemmqlk2 |hashdollar-PMED4.PMM0817|

|hashdollar-prochlorococcus_marinus_mit9313|)

|hashdollar-prochlorococcus_marinus_mit9313|)

(resolve 5425 :code-for-gene-in-organism-code)

Answer (or (snark::answer-- (list |hashdollar-PMED4.PMM0817|))

(defn-of-differentially-regulated

|hashdollar-PMED4.PMM0817| light

|hashdollar-S6803.ssr2595|

|hashdollar-synechocystis_pcc6803|)))

(Row 5428

snark-lisp:false

(resolve 5427 :code-for-gene-has-ortholog-in-organism)

Answer (or (snark::answer-- (list |hashdollar-PMED4.PMM0817|))

(defn-of-differentially-regulated

|hashdollar-PMED4.PMM0817| light

|hashdollar-S6803.ssr2595|

|hashdollar-synechocystis_pcc6803|)))

)

; Summary of computation:

; 5454 formulas have been input or derived (from 3629 formulas).

; 5428 (100%) were retained. Of these,

; 70 ( 1%) were simplified or subsumed later,

; 5358 (99%) are still being kept.

;

; Run time in seconds excluding printing time:

; 1.560 3% Resolution (3628 calls)

; 0.090 0% Factoring (3620 calls)

; 0.000 0% Condensing (22 calls)

; 40.210 75% Forward subsumption (5425 calls)

; 5.130 10% Backward subsumption (5424 calls)

; 0.000 0% Clause clause subsumption (86 calls)

; 2.500 5% Forward simplification (5451 calls)

; 0.020 0% Backward simplification (5428 calls)

; 0.180 0% Ordering (11413 calls)

; 0.020 0% Sortal reasoning (262 calls)

; 3.660 7% Other

; 53.370 Total

;

(SNARK::ANSWER-- (LIST #$PMED4.PMM0817))

> ((SNARK::QUESTION-- (ADAPTIVE-GENE ?GENE MED4 LIGHT)) NIL (SNARK::ANSWER-- (LIST #$PMED4.PMM0817)))
